# Novel β-Hairpin Peptide from Marine Polychaeta with a High Efficacy against Gram-Negative Pathogens

**DOI:** 10.3390/md20080517

**Published:** 2022-08-13

**Authors:** Victoria N. Safronova, Ilia A. Bolosov, Roman N. Kruglikov, Olga V. Korobova, Eugenia S. Pereskokova, Alexander I. Borzilov, Pavel V. Panteleev, Tatiana V. Ovchinnikova

**Affiliations:** 1 M. M. Shemyakin & Yu. A. Ovchinnikov Institute of Bioorganic Chemistry, The Russian Academy of Sciences, Miklukho-Maklaya Str., 16/10, 117997 Moscow, Russia; 2 State Research Center for Applied Microbiology & Biotechnology (SRCAMB), 142279 Obolensk, Russia; 3 Department of Bioorganic Chemistry, Faculty of Biology, Lomonosov Moscow State University, 119234 Moscow, Russia

**Keywords:** antimicrobial peptide, polychaeta, BRICHOS domain, abarenicin, mice model, antimicrobial resistance, antibiofilm activity

## Abstract

In recent years, new antibiotics targeting multidrug resistant Gram-negative bacteria have become urgently needed. Therefore, antimicrobial peptides are considered to be a novel perspective class of antibacterial agents. In this study, a panel of novel BRICHOS-related β-hairpin antimicrobial peptides were identified in transcriptomes of marine polychaeta species. Two of them—abarenicin from *Abarenicola pacifica* and UuBRI-21 from *Urechis unicinctus*—possess strong antibacterial potential in vitro against a wide panel of Gram-negative bacteria including drug-resistant strains. Mechanism of action assays demonstrate that peptides disrupt bacterial and mammalian membrane integrity. Considering the stronger antibacterial potential and a low ability of abarenicin to be bound by components of serum, this peptide was selected for further modification. We conducted an alanine and arginine scanning of abarenicin by replacing individual amino acids and modulating hydrophobicity so as to improve its antibacterial potency and membrane selectivity. This design approach allowed us to obtain the Ap9 analog displaying a high efficacy in vivo in the mice septicemia and neutropenic mice peritonitis models. We demonstrated that abarenicin analogs did not significantly induce bacterial resistance after a four-week selection experiment and acted on different steps of the biofilm formation: (a) killing bacteria at their planktonic stage and preventing biofilm formation and (b) degrading pre-formed biofilm and killing embedded bacteria. The potent antibacterial and antibiofilm activity of the abarenicin analog Ap9 with its high efficacy in vivo against Gram-negative infection in mice models makes this peptide an attractive candidate for further preclinical investigation.

## 1. Introduction

The increasing problem of antibiotic resistance becomes a serious challenge for public health worldwide [1]. A growing number of drug-resistant bacterial species require considerable effort if one is to find new classes of antibiotics with radically different mechanisms of action. One of the promising alternatives is the use of antimicrobial peptides (AMPs), which are produced by the majority of multicellular organisms as molecular components of innate immunity system. Although many AMPs have reached clinical trials, none of them have been approved yet by the US Food and Drug Administration (FDA) due to several limiting factors: systemic toxicity, proteolytic degradation, and short half-life. To solve these problems, a huge number of structure-function studies of both natural and synthetic peptides were carried out with a view to design molecules with a low toxicity and a high antibacterial activity [2].

In this regard, the family of β-hairpin peptides termed arenicins—isolated from coelomocytes of marine polychaeta *Arenicola marina*—attracts much attention. There are three members of this family: arenicin-1, -2 containing one disulfide bridge and arenicin-3 with two disulfide bonds [3,4,5,6]. Natural isoforms of arenicins exhibit a pronounced antimicrobial activity against Gram-negative and Gram-positive bacteria as well as towards yeasts and pathogenic fungi. Notably, it has been found that arenicin-3 had a high serum-binding ability and like other arenicins possessed a marked toxicity towards mammalian cells, but was the most potent against various pathogens [7]. Structure-based design of arenicin-3 analogs allowed to select a variant, abbreviated as AA139, which retained a strong antibacterial activity and had a reduced cell toxicity compared to the original peptide. Furthermore, AA139 displayed in vivo efficacy in infection models of peritonitis, urinary tract, and pneumonia [8].

Here, we found 11 novel arenicin-like peptides in different polychaeta species. The peptides are synthesized as *C*-terminal parts of corresponding precursor proteins containing the BRICHOS domain. Among them are β-hairpin peptides from the Pacific lugworm *Abarenicola pacifica* (named abarenicins) and from the marine spoon worm *Urechis unicinctus* (designated as UuBRI-21). The peptides were obtained in a bacterial expression system and studied. With the purpose of enhancing antimicrobial potential and minimizing toxicity effects, we designed a panel of abarenicin analogs by substituting selected hydrophobic amino acid residues with arginine and/or alanine. In particular, we examined the ability to damage bacterial membranes and induce bacterial resistance, proteolytic stability in blood serum, and antibiofilm activity of the obtained peptides. By means of biological screening of all analogs, the compounds with more potent antibacterial and fewer toxic properties were selected for in vivo investigation of efficacy in *Escherichia coli* infection mice models.

## 2. Results and Discussion

### 2.1. Identification of Novel β-Hairpin AMPs

**Figure 1:**
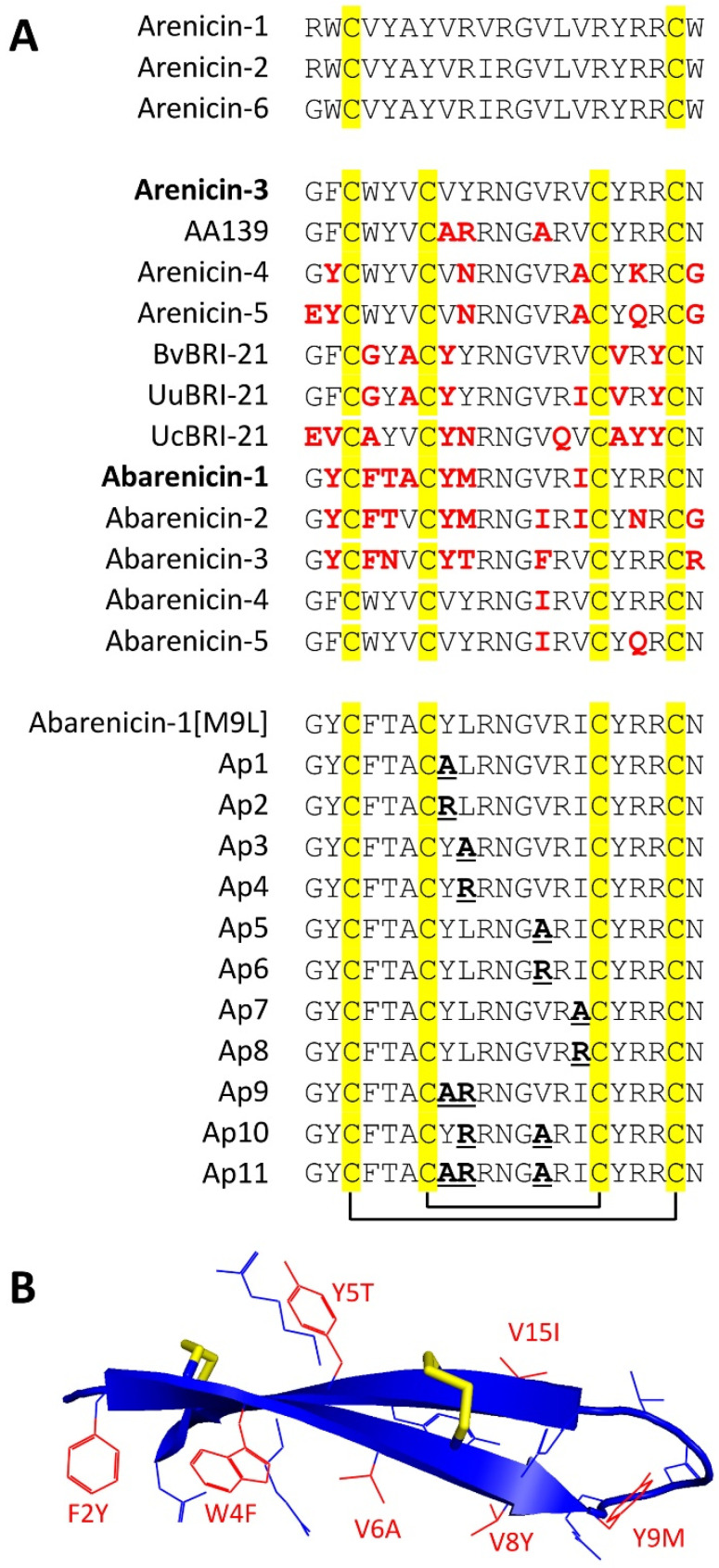
(**A**) Amino acid sequence alignment of the novel BRICHOS-related peptides and modified analogs of abarenicin-1 with known arenicins. Variable amino acid residues in the peptides as compared to arenicin-3 are colored in red. Amino acid substitutions in the analogs Ap1–11 as compared to abarenicin-1(M9L) are bold and underlined. (**B**) Spatial structure of arenicin-3 (PDB 5V0Y, the model was visualized with the PyMOL software). Localization of seven different amino acid residues in the sequence of abarenicin-1 as compared to arenicin-3 are colored in red.

In this study, we performed de novo assembly of transcriptomes of several polychaeta species mentioned below using the NCBI Sequence Read Archive (SRA) data and found 11 sequences of target BRICHOS-related arenicin-like peptides stabilized by one or two disulfide bonds (Figure 1A). First, we found three peptides in *Arenicola marina* (NCBI accession number SRX1015734) named arenicin-4, -5, and -6. Arenicin-4, designated as ANN4, was described earlier when a transcriptomic profiling of coelomocytes of lugworm *A. marina* was performed [9]. Arenicin-5 has the highest homology with arenicin-4 whereas arenicin-6 is an analog of anenicin-2 with the amino acid substitution of *N*-terminal arginine residue for glycine. We also found transcripts coding five novel arenicin-like peptides in the Pacific lugworm *Abarenicola pacifica* (SRX7658526) that is closely related to *A. marina*. The peptides were named abarenicins. Abarenicin-1 has seven amino acid substitutions as compared to arenicin-3. Interestingly, most alterations are located in the *N*-terminal strand and make the peptide less hydrophobic (Figure 1B). Abarenicin-2 and -3 also have a set of substitutions in both *N*- and *C*-terminal strands as compared to arenicin-3 (Figure 1A). In contrast, abarenicin isoforms 4 and 5 have one (V13I) and two (V13I, R18Q) amino acid substitutions, respectively, as compared to arenicin-3. Three additional BRICHOS-related AMPs were predicted in the spoon worms *Bonellia viridis* (SRX1024222), *Urechis unicinctus* (SRX4526081), and *U. caupo* (SRX6205158). This group of marine animals is evolutionary related to *Capitellidae* [10], which are known to synthesize the β-hairpin AMP capitellacin [11]. Notably, the precursors of these AMPs are transmembrane proteins consisting of three parts: an *N*-terminal short piece, a transmembrane helix, and a *C*-terminal part that includes BRICHOS domain and AMP sequence (Figure S1). This is in a sharp contrast to preproarenicins and preproabarenicins which contain *N*-terminal signal sequences. The peptides were named according to their origin and number of amino acid residues as BvBRI-21, UuBRI-21, and UcBRI-21. The Xaa-Lys-Arg motif preceding a putative propeptide cleavage site of the majority of the found peptides indicated that a precursor is probably activated by furin proteases, which is common for other BRICHOS-related AMPs.

### 2.2. Recombinant Expression and Biological Activity of the Novel β-Hairpin AMPs

During the first stage, we obtained two novel AMPs: abarenicin-1 (M9L) (herein entitled ‘abarenicin’) and UuBRI-21. Both peptides have a low homology with known arenicins. The M9L substitution was made to prevent fragmentation of abarenicin by cleavage of the fusion protein at the methionine residue using cyanogen bromide. Heterologous expression of the peptides was performed in the bacterial system as described earlier [12]. Briefly, the peptides were fused with thioredoxin A (M37L) to facilitate the correct disulfide bond formation and mask toxic effects of peptides. All of the fusion proteins were expressed in *E. coli* BL21 (DE3) cells and the clarified lysates were purified by metal chelate chromatography under denaturing conditions on the column packed with Ni Sepharose. Target peptides were obtained by cleavage with cyanogen bromide and reverse-phase high performance liquid chromatography (RP-HPLC) was used for the final purification step. MALDI mass spectrometry analysis of the main fractions showed that the measured values of monoisotopic *m*/*z* matched well with the calculated molecular masses of protonated ions (M + H)^+^ of corresponding peptides indicating formation of two disulfide bonds (Table S1). The final yields of the recombinant peptides were 4.4–11.2 mg per 1 L of the culture.

Initially, antimicrobial activity of abarenicin and UuBRI-21 was evaluated against the reference strain of *E. coli* (ML-35p) under various conditions (Table 1). Minimal inhibitory concentrations (MICs) were determined via two-fold serial dilutions in 96-well polystyrene plates. Serial dilutions were made in 0.1% bovine serum albumin (BSA) to minimize the absorption of peptides on the surface of plate wells. Mueller–Hinton broth (MHB) supplemented with 0.9% NaCl was used as the standardized medium. Moreover, it was interesting to examine how the presence of fetal bovine serum (FBS) in the medium impeded activity of peptides and to evaluate an effective concentration expected in experiments with the use of in vivo models. All the peptides had similar MIC values of 0.25 µM against *E. coli* ML-35p in MHB with 0.9% NaCl. As excepted, an antibacterial activity was suppressed in the presence of 25% FBS and led to a 4-fold reduction of the activity of abarenicin and AA139. The most hydrophobic peptide UuBRI-21 according to estimated RP-HPLC retention time (28.48 min, Table S1) demonstrated the highest affinity to components of FBS resulting in the reduced 16-fold activity compared to abarenicin and AA139. We assume that a key role in a significant decrease in activity of UuBRI-21 was associated with a higher degree of hydrophobicity and could be caused by binding to FBS proteins. Considering the received data in FBS-supplemented microdilution assay, abarenicin was chosen as a template to enhance selectivity against Gram-negative bacteria.

### 2.3. Abarenicin Analogs Display a Potent Antibacterial Activity against Gram-Negative ESKAPE Pathogens

In this study, we systematically examined the importance of each hydrophobic amino acid in the β-turn region in a search for therapeutically valuable analogs of abarenicin (Figure 1A). The presence of hydrophobic residues is necessary for membrane permeabilization. However, a high degree of hydrophobicity leads to a loss of an antibacterial selectivity and toxicity towards host cells [13]. Here, we produced an alanine and arginine scanning series by replacing individual amino acid residues independently. It was shown that amino acids substitutions in the β-turn region of arenicins and tachyplesins for these residues often led to an enhance of the selectivity due to a decrease in cytotoxic properties of the molecule [4,5,10,14]. No cysteine residues were mutated, in order to maintain the integrity of the peptide backbone and the secondary structure that is required for manifestation of an antimicrobial activity.

**Figure 2:**
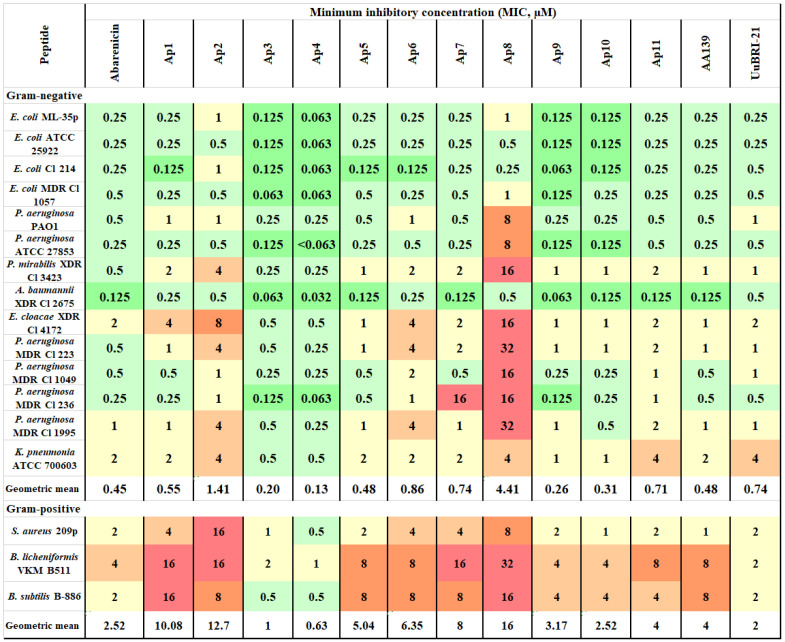
Antibacterial activity of the peptides.

Screening of an antimicrobial activity of the peptides was assessed using a two-fold serial microdilution assay that determines MICs against a panel of opportunistic Gram-positive and Gram-negative bacteria. As follows from the color gradation in Figure 2, the peptides predominantly kill Gram-negative bacteria, including MDR and XDR strains. Abarenicins and UuBRI-21 demonstrated a comparable level of activity. Notably, these marine peptides are salt-insensitive antimicrobial agents. Their potential recovers in medium containing physiological concentration of sodium chloride, which reduces an activity of many natural AMPs [15]. Substitutions of either Y8 or I15 residue for arginine in abarenicin structure resulted in a significant decrease in the activity against the tested bacteria. Compared with abarenicin, the greatest antimicrobial potential was shown by Ap4 (L9R) against Gram-negative bacteria (GM value of 0.13 µM). However, the most potent abarenicin analog displayed a strong hemolytic activity. We also designed the analogs of the variant Ap4 bearing additional substitution(s) of Tyr8 and/or Val13 for a less hydrophobic alanine residue to improve cell selectivity and retain potent antimicrobial properties. The modulation of hydrophobicity at positions 8 (analog Ap9) and 13 (analog Ap10) resulted in a reduced antimicrobial activity in contrast with Ap4, but these compounds were still more effective against Gram-negative bacteria than the wild type abarenicin (GM values 0.26/0.31, respectively, vs. 0.45). The analog Ap11 with two amino acid substitutions (Y8A, V13A) showed a marked, at least 5-fold reduction in the antimicrobial activity compared to Ap4. Importantly, additional modifications in the structure of the variant Ap4 led to analogs with a decreased hemolytic potency and a retained antibacterial activity of abarenicin. Figure 2 shows that all analogs excluding Ap2 and Ap8 display a broad spectrum of activity comparable with that or even higher than that of abarenicin.

### 2.4. The Analogs of Abarenicin Selectively Target Bacterial Membranes

**Figure 3:**
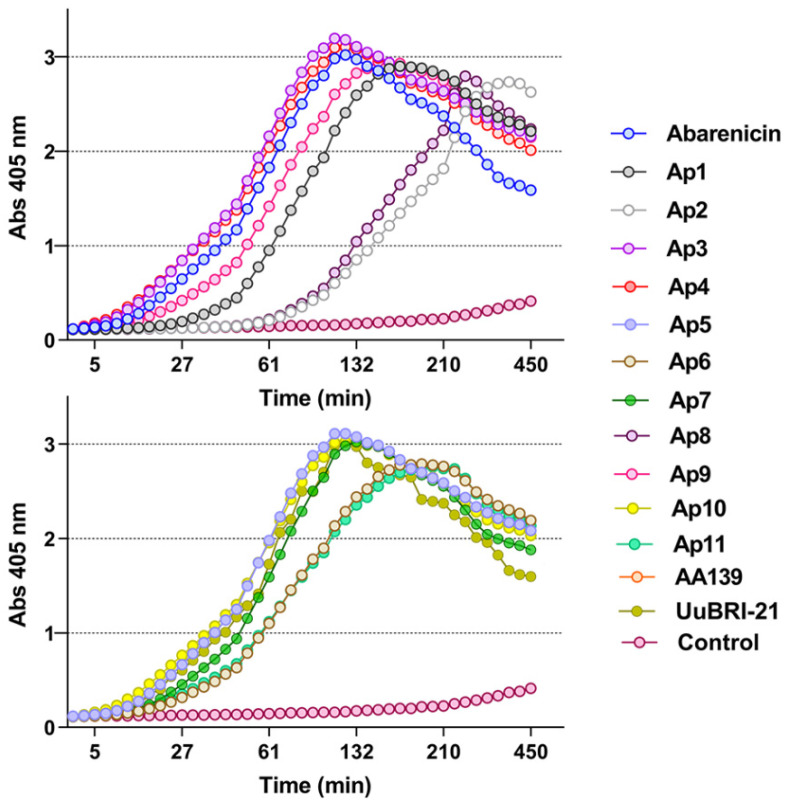
Kinetics of changes in the *E. coli* ML-35p cytoplasmic membrane permeability measured with the use of ONPG (OD_405_) hydrolysis caused by the peptides at a concentration of 4 µM. Control measurements were carried out without antimicrobial agents. Three independent experiments were performed, and the curve pattern was similar.

Many AMPs kill bacteria by disrupting bacterial membranes, which results in content leakage or cell membrane lysis. One of the obstacles for their development as new membrane-targeting antibiotics is cytotoxic and hemolytic effects toward mammalian cells through non-selective membrane damage. Thus, an ability of the peptides to penetrate the cytoplasmatic membrane of *E. coli* ML-35p was examined using the chromogenic marker o-nitrophenyl-β-D-galactopyranoside (ONPG). The ML-35p strain is characterized by constitutive synthesis of β-galactosidase in the bacterial cytoplasm and the absence of lactose permease. An increase in the permeability of the cytoplasmic membrane was estimated spectrophotometrically at 405 nm by the reaction product accumulation upon cleavage of the substrate ONPG by β-galactosidase [11]. As shown in Figure 3, all compounds exhibited pronounced membranolytic effects, as illustrated by an increased absorbance of the solution due to appearance of the colored product of ONPG hydrolysis. Ap2 and Ap8 also destroy the integrity of the *E. coli* membrane, but demonstrate slower permeability kinetics, which corresponds well with their lowest antibacterial potency against Gram-negative bacteria (GM value of 1.41 and 4.41, respectively).

**Figure 4:**
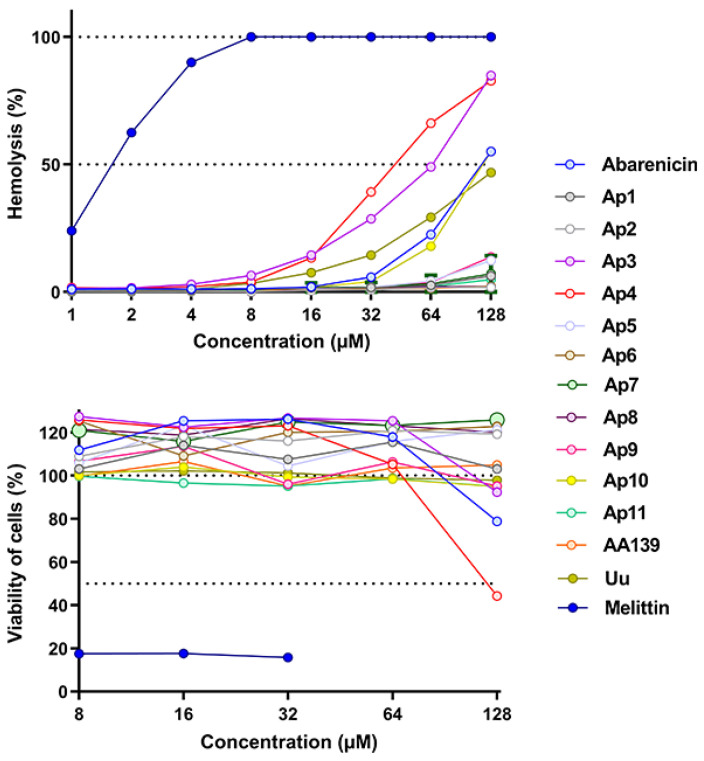
(**A**) Hemolytic effect on human red blood cells after 2 h incubation (hemoglobin release assay). (**B**) Cytotoxicity toward transformed human embryonic kidney cells after 20 h incubation (MTT-assay). The positive control used was 0.1% Triton X-100 and sterile PBS as a negative control. Melittin was used as an additional positive control. The data are presented as the mean ± SD of three independent experiments.

It is rather difficult to predict the contribution of amino acid residues to the antibacterial activity and selectivity of the peptide. It is generally accepted that a decrease in the hydrophobicity of the peptide would reduce hemolytic properties compared to a parent peptide [16]. Toxicity of the peptides toward eukaryotic cells was determined by using red blood cells (hRBCs) and transformed human embryonic kidney cells (HEK293T). In Figure 4A, it was demonstrated that the native peptides (abarenicin and UuBRI-21) exerted a moderate hemolytic activity, and the maximum level of lysis was 55% and 47% at a concentration of 128 µM, respectively. Thus, the most active and less hydrophobic peptides Ap3 и Ap4 than abarenicin also target both bacterial and mammalian membrane and their ability to lyse hRBCs is stronger. In our study, it is clearly seen that hydrophobicity and selectivity did not have a direct correlation. Other abarenicins, except for Ap10, did not display toxic effects and lysed less than 3–4% at the concentration of 64 µM.

Next, an MTT test was carried out as an additional analysis for assessing the toxicity of the peptides (Figure 4B). Moderate eukaryotic damage was observed only for abarenicin and its analog Ap4, however most peptides were not able to cause cytotoxic effect against HEK293T even at the highest concentration (128 µM). In contrast to abarenicin, UuBRI-21 lacked cytotoxic activity against mammalian cells at concentrations up to 128 µM, which may be due to interactions with proteins of serum added to the medium. In our previous study, a similar suppression of the cytotoxicity of arenicin-1 in the FBS presence was revealed [15]. Considering the antibacterial activity and toxicity of other analogs, Ap9 with a strong antimicrobial activity and an insignificant toxicity toward human erythrocytes and completely non-toxic Ap11, whose activity profile is slightly inferior to AA139, were selected for our further investigation as drug candidates.

### 2.5. Stability of Abarenicin to Degradation by Serum Proteases

**Figure 5:**
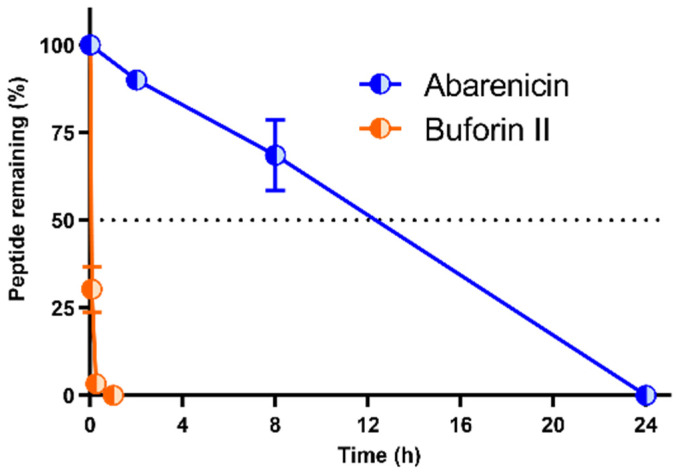
Stability profile of abarenicin and buforin II in serum. The peptides were incubated in 25% fresh human serum for 0–24 h at 37 °C and their relative concentrations were determined by RP-HPLC on the Symmetry 300 C18 column. The height of the UV absorbance peak at 214 nm on the RP-HPLC trace was used to calculate a percentage of the remained peptide. The data are presented as the mean ± SD of two independent experiments.

The development of antimicrobial peptides for systemic application is often limited by their low stability as a result of rapid proteolytic degradation. Design strategies to improve resistance of peptides to serum proteases include peptide modifications, such as acetylation of the *N*-terminus, amidation of the *C*-terminus, replacement with unnatural amino acids (mainly with D-amino acids), PEGylation, and head-to-tail cyclization [17]. Peptide stability in blood is often studied in vitro by mixing the drug and serum or plasma, allowing for the identification of their cleavage sites. In this study, resistance of abarenicin and the linear AMP buforin II to enzymatic degradation were determined using RP-HPLC; MALDI-TOF MS was used to identify the fractions produced. A degradation rate was analyzed in 25% fresh human serum for 0–24 h at 37 °C. The results shown in Figure 5 demonstrated that more than 90% of abarenicin remained intact after 2 h, whereas buforin II was quickly degraded with half-lives of less than 5 min. The main product of abarenicin proteolysis was the peptide without the *N*-terminal glycine, and its antimicrobial activity was also determined. This form was shown to possess a decreased antimicrobial activity against *E. coli* ML-35p/ATCC 25,922 (MIC of the truncated peptide amount of 1/2 µM, respectively, compared to MIC of the unmodified one of 0.25/0.25 µM). These data highlight the importance of the *N*-terminal Gly residue for an enhanced antimicrobial activity and demonstrate that two disulfide bridges provide the rigidity of the abarenicin structure and its optimal stability in blood serum.

### 2.6. Abarenicins Do Not Induce a Strong Bacterial Resistance

**Figure 6:**
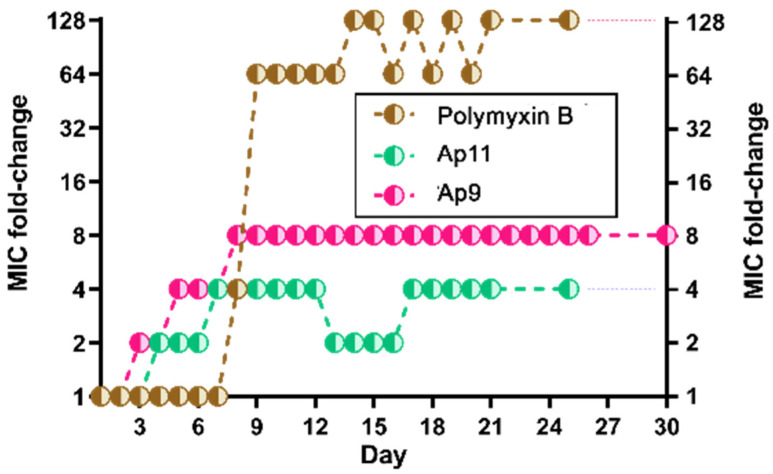
Serial passages for resistance induction by abarenicins (Ap9 and Ap11) and antibiotic polymyxin B of *E. coli* MDR Cl 1057 in 0.9% NaCl-enriched Mueller–Hinton medium. Initial MIC values were Ap9 (0.125 µM), Ap11 (0.25 µM) and polymyxin B (0.125 µM).

A major health problem that poses significant challenges is the widespread emergence of bacterial resistance to conventional antibiotics. Since AMPs are considered to be new generation of antimicrobial agents, it is necessary to more fully study a possible mechanism of development of bacterial resistance to them. It is believed that the occurrence of resistance development to AMPs is unlikely since their mechanisms of action differ from those of traditional antibiotics. A wide panel of different laboratory assays are available to isolate drug-resistant mutants of bacteria. One of them involves a series passage with suspension of a test strain and liquid medium containing an antibacterial agent at concentrations near the MIC, which progressively increases during the experiment until resistant mutants are enriched. Bacteria that grew at the highest concentration of drug are used in the final step of the test that determines the final MIC value [18]. Next, the resistance of *E. coli* MDR Cl 1057 to abarenicins (Ap9 and Ap11) was induced using the broth microdilution test (Figure 6). This clinically isolated strain carried two well-characterized mutations in gyrA (S83L and D87N) causing a high-level fluoroquinolone resistance, as well as a higher-than-normal spontaneous mutation rate [19]. Approved by WHO as the main drug for the treatment various infections caused by Cram-negative bacteria including multidrug-resistance strains, polymyxin B was taken as the reference agent [20]. This experiment indicated that abarenicins induced a low-level stable resistance of *E. coli* with MICs up to 4-fold (for Ap11) and 8-fold (for Ap9) higher than those of the wild-type strain. The following subculturing with 3 passages (days) in the absence of the peptide showed that their final MICs were not changed. Notably, polymyxin B rapidly induced a stable resistance and displayed a 128-fold increase in the MIC value, which corresponded well to our previous results [21]. An important factor is that abarenicins do not induce a stable resistance of *E. coli* MDR Cl 1057 compared to the membrane-active polymyxin B, which has a similar mechanism of action. A small change in MIC may be associated with modification of definite targets on the outer membrane surface or any changes in lipid composition of bacterial cell membranes, which might decrease affinity to abarenicins. The whole genome sequencing of these strains was performed further to identify genes involved in the development of this stable low-level bacterial resistance.

### 2.7. Abarenicins Prevent Biofilm Formation and Kill Embedded Cells Inside Biofilms Formed by P. aeruginosa including Its Drug-Resistance Clinical Isolates

**Figure 7:**
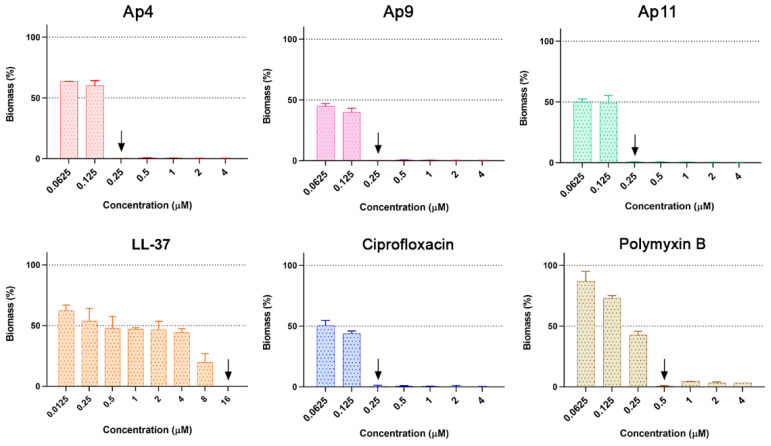
Impact of abarenicins (Ap4, Ap9, Ap11), the human AMP LL-37, and conventional antibiotics ciprofloxacin and polymyxin B on biofilm formed by *P. aeruginosa* PAO1. Masses of biofilms were evaluated by dying with 0.1% CV. Sterile water was used as a solvent for serial dilutions of peptides. Down arrow is MIC of peptides in cases of a significant difference. *p* ≤ 0.0001 not shown.

**Figure 8:**
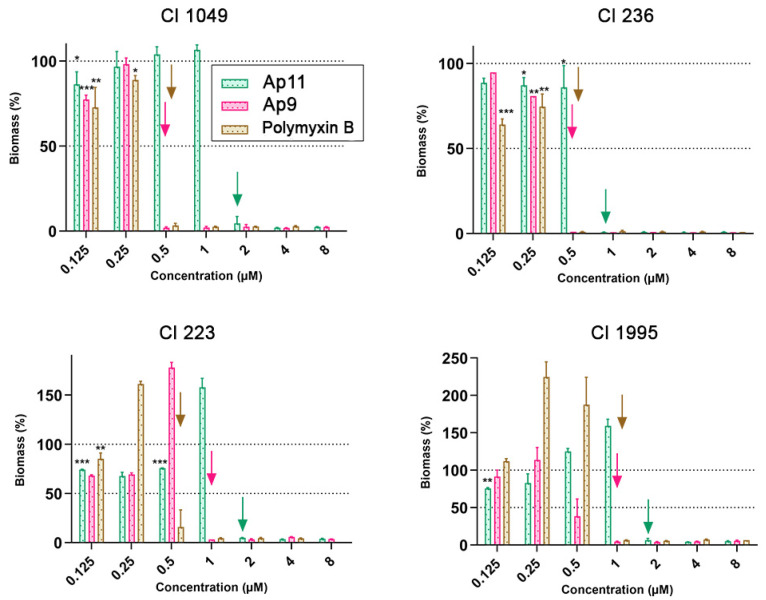
Anti-biofilm activity of Ap9, Ap11, and Polymyxin B against clinical isolates of *P. aeruginosa* (Cl 1049, Cl 236, Cl 223, and Cl 1995). Down arrow is MIC of peptides, asterisks show statistically significant differences between the treated and untreated biofilms (* *p* ≤ 0.05, ** *p* ≤ 0.01, *** *p* ≤ 0.001, in cases of a significant difference, *p* ≤ 0.0001 not shown).

Some microorganisms possess a unique ability to protect themselves from harmful environmental effects. Such an adaptive and survival mechanism is mediated by biofilms, which constitutes the community of sessile bacteria embedded within the self-produced extracellular polymeric substance (EPS) that defends them from chemical, biological, and physical stress; it also diffuses foreign invaders. Besides the fact that EPS decreases concentration of antibiotics, bacteria inside biofilm are characterized by a reduced rate of cell divisions, an increased expression of stress response genes, an occurrence of persistent cells, and exchange of resistance genes leading to a broader spread of tolerant biofilm-growing bacteria [22]. For the treatment of biofilm-associated infections a high concentration of antibiotics is required that also increases toxicity toward human cells. Antimicrobial peptides are able to act on certain stages of biofilm formation and various molecular targets and are considered as promising candidates for the development of new antibiofilm agents. In addition, the bactericidal mechanism of action of AMPs makes them attractive for the treatment of severe biofilm infections [23]. Several AMPs were tested on ability to impair bacterial biofilms. For instance, the commonly investigated human peptide LL-37 can kill planktonic bacteria while also being capable of acting on sessile bacteria of *P. aeruginosa* by downregulating two quorum-sensing systems [24]. *P. aeruginosa* represents one of the most socially important bacteria involved in nosocomial infections, which cause pneumonia, cystic fibrosis, urinary tract infections, and medical device-associated infections [25]. Its high tendency to colonize both abiotic and biotic surfaces makes it a suitable in vitro system for anti-biofilm studies. Previously, the biofilm-forming ability of *P. aeruginosa* PAO1 on wells of plate was estimated using the crystal violet staining method [26]. Considering the confirmed data, PAO1 was selected as a reference strain. In this research, we set a goal to compare abarenicins (Ap4 with the highest GM value, and selective compounds Ap9, Ap11), clinically used antibiotics polymyxin B and ciprofloxacin, and natural well-studied host-defense cathelicidin LL-37 for antibiofilm properties. Bacterial biomass amount was evaluated using the standard crystal violet assay over a 24 h incubation period with each drug. As shown in Figure 7, all compounds exhibited strong activity, completely inhibiting the biofilm formation of *P. aeruginosa* PAO1 at MIC in a concentration-dependent manner. This was in sharp contrast to other known β-hairpin AMPs, which have been shown to stimulate biofilm production at sub-inhibitory concentrations [26]. Ap4, Ap9, Ap11, and ciprofloxacin had a similar antibiofilm potency, however Ap9 was more effective in biofilm prevention at sub-inhibitory concentration. LL-37 and polymyxin B demonstrated a modest inhibition of biofilm, and LL-37 was shown to reduce biofilm growth by 40% at a concentration of 1/128 MIC, which is consistent with previous findings [27]. Apart from antibiofilm activity against the PAO1 strain, abarenicins inhibit biofilm formation by different clinical isolates of *P. aeruginosa,* that are uropathogenic strains isolated from patients with kidney stone disease (Figure 8). Expectedly, our results indicated that ciprofloxacin had no anti-biofilm effect on these fluoroquinolone-resistant clinical isolates at concentrations up to 32 µM and also caused a strong stimulation of biofilm growth. Abarenicins and polymyxin B at sub-inhibitory concentrations also stimulated biofilm formation of clinical isolates No. 223 and No. 1995. The same effect on biofilm of *P. aeruginosa* was demonstrated for aminoglycoside and tetracycline antibiotics [28,29]. The overall ability of abarenicins to prevent biofilm formation makes them a perspective agent to combat biofilm-related infection caused by *P. aeruginosa*, though special attention should be directed at the selection of an optimal concentration of peptides to avoid stimulation of biofilm growth.

**Figure 9:**
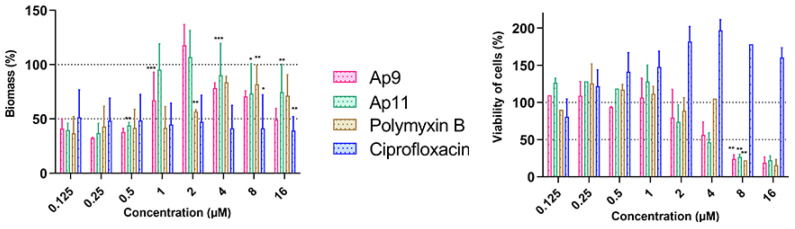
Eradicating effect of abarenicins, polymyxin B, and ciprofloxacin on *P. aeruginosa* PAO1 mature biofilms. Biofilm biomasses was assessed using the crystal-violet staining technique (left panel). Biofilm viability was measured by tetrazolium salt reduction (right panel). Values are presented as means ± the standard deviation (SD) of three independent experiments. Asterisks indicate statistically significant differences between the treated and untreated biofilms.

Another challenge in fighting bacterial biofilms is the difficulty of destroying them once they are already established. EPS provides a physical barrier and escape mechanism from treatment and, if it is degraded, biofilm dispersal and enhanced access of antibacterial agents for bacteria inside biofilm might be observed [30]. Then, an ability of the peptides to affect mature biofilms of *P. aeruginosa* PAO1 was assessed (Figure 9). The pre-formed biofilms were incubated with drugs at concentrations several times higher than their MICs for a period of time that is relevant to β-hairpin peptide pharmacokinetics [8] and stability of abarenicin in blood serum (Figure 5). The biomass of biofilm was quantified by crystal-violet staining. A decrease of biomasses after 2-h exposure was observed at sub-inhibitory concentrations. Interestingly, no correlation was found between a concentration of each compound and degree of EPS degradation. Biofilm consists of EPS and bacteria are immersed in it. As crystal-violet staining does not allow to distinguish between living and dead cells, we performed a metabolic dye reduction using MTT assay. Abarenicins and polymyxin B showed a visible reduction in the number of viable bacteria, whereas ciprofloxacin was not able to impair bacteria inside biofilm. A treatment with 16 µM of Ap9, Ap11 or polymyxin B resulted in a minimum residual (about 20%) viability of bacteria. Thus, abarenicins possess a variety of active biofilm defense mechanisms that can potentially be clinically used. However, further research is needed to clarify the molecular mechanism of antibiofilm action for these peptides.

### 2.8. In Vivo Efficiency of the Novel Peptides

**Figure 10:**
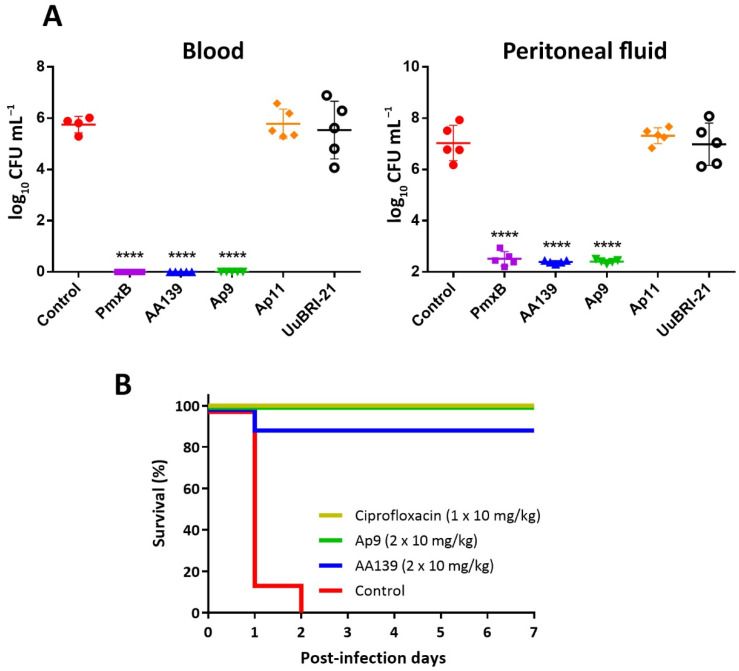
In vivo efficacy studies. (**A**) The mice peritonitis model. Neutropenic BALB/c mice (*n* = 5) infected i.p. with *E. coli* 3421/E19 were treated i.v. with a single dose of AMPs (10 mg/kg) or control antibiotic polymyxin B (PmxB, 5 mg/kg). CFU counts were determined in blood and peritoneal fluid 5 h after treatment. Horizontal bars indicate means ± SD. Asterisks indicate statistically significant differences with control (**** *p* ≤ 0.0001). (**B**) Survival rates of BALB/c mice (*n* = 8) infected i.p. with *E. coli* ATCC 25922 (10^6^ bacteria in the presence of 2.5% mucin). Ciprofloxacin (the single dose of 10 mg/kg) and saline were used as the positive and negative controls, respectively. Two other groups received abarenicin analog Ap9 or AA139 each administered i.p. two times (1 and 4 h post-infection) at a dose of 10 mg/kg. Mice were controlled once daily for their health status during 7 post-infection days.

An appropriate balance of a high antibacterial activity and a low cytotoxicity, as well as the retention of an antimicrobial activity level in the presence of serum (a 4-fold reduction of MIC value against *E. coli* ML-35p), suggested that the modified analogs Ap9 and Ap11 could be considered as lead candidates suitable for testing in mice models of Gram-negative infections. Moreover, we also selected UuBRI-21 as a compound that lacked any cytotoxicity against adherent mammalian cells in vitro. First, we evaluated the in vivo efficacy of the peptides against the virulent *E. coli* 3421/E19 strain, using a single intravenous (i.v.) dose of 10 mg per kg of body weight in a neutropenic mice peritonitis model (Figure 10A). Immune-deficient mice were inoculated intraperitoneally (i.p.) with bacterial suspension. Antimicrobial agent was administered 1 h post-infection, and colony counts in blood and peritoneal fluid (PF) were determined 5 h later. Despite all the peptides tested had similar MIC values of 0.5–1 µM against *E. coli* 3421/E19 in MHB medium, only Ap9 and AA139 (served as the control AMP) were effective in this animal model. Administration of these peptides, as well as of the control antibiotic polymyxin B, resulted in a complete reduction of bacterial burden in mice blood, and substantial (~5-log) reduction of the CFU level in PF. Our results are comparable to those obtained earlier with AA139 using similar neutropenic mice model infected with *E. coli* [8]. Surprisingly, the abarenicin analog Ap11 having similar structural modification and in vitro biological activity profile as compared with AA139 was ineffective in the peritonitis model. These quite unpredictable results point at the significance of in vivo screening of peptide drug candidates using small-number animal groups (*n* = 3–5) prior to preclinical studies of these compounds. The lack of effectivity of UuBRI-21 could result from its higher ability to be inactivated by serum components, as is shown in Table 1.

Then, we examined an efficacy of AMPs in the mice septicemia model using 8 animals in a group (Figure 10B). To estimate animal survival rates, we used the selected abarenicin analogs Ap9 and AA139, and applied each of them two times (10 mg/kg in each injection) in the first day after infection. Intraperitoneal infection of BALB/c mice with *E. coli* ATCC 25922 in the presence of mucin resulted in the death of all mice within two days if treated using the vehicle control (saline) and survival of all mice when treated with 10 mg/kg ciprofloxacin. Here, we demonstrated a therapeutic efficacy of 100% and 87.5% when applying the double dose of the abarenicin analog Ap9 and the arenicin-3 analog AA139, respectively, after one-week experiment. A high CFU burden in mice from the negative control group was verified. Expectedly, we also did not identify *E. coli* in spleen after euthanizing of all survived animals. These data suggest that the modified variants of marine polychaeta β-hairpin AMPs are promising anti-Gram-negative antimicrobials.

## 3. Materials and Methods

### 3.1. Transcriptome Assembly

The raw data for all samples was obtained from NCBI Sequence Read Archive (SRA). The data was converted to FASTQ format with FASQ-dump utility of SRA-toolkit and then the quality of reads was assessed using FastQC. The raw reads for each sample were filtered to remove low quality reads and adapter sequences using Trimmomatic software (v.0.38) with following parameters: ILLUMINACLIP:2:30:10, LEADING:30, TRAILING:30, SLIDINGWINDOW:10:25. The trimmed reads were then filtered from ribosomal RNA by aligning them to the SILVA database (release 138.1) using bowtie2 (v.2.3.5.1). Unaligned reads were used to assemble transcriptome with the Trinity pipeline (v.2.9.1); they had a minimum contig length of 200 bp. Assembled transcriptomes were then directly translated and converted into protein blast databases. The target β-hairpin AMPs were identified by blasting known arenicin sequences and conservative regions of the BRICHOS domain (Figure S1) against obtained databases with BLASTP algorithm. The raw data including species names, accession numbers, total amount of the sequencing reads, and sequencing platform are provided in the Table S2. The nucleotide sequence encoding preproUcBRI-21 was found by direct search in SRX6205158 project using BTASTN instrument and UuBRI-21 transcript as a query.

### 3.2. Expression and Purification of the Antimicrobial Peptides

The recombinant plasmids for expression of arenicin-like peptides were constructed with the use of pET-based vector as described previously [12]. The nucleotide sequences were designed on the basis of *E. coli* codon usage bias. The expression cassette was composed of the T7 promoter, the ribosome binding site, and the sequence encoding the chimeric protein that included His-tag, the *E. coli* thioredoxin A (M37L) (TrxL), methionine residue, and a mature peptide. *E. coli* BL21 (DE3) cells were transformed with the corresponding plasmids and grown in lysogeny broth (LB) containing 20 mM glucose, 1 mM MgSO_4_, 100 µg/mL of ampicillin up to OD_600_ 0.7÷1, and then were induced with isopropyl β-D-1-thiogalactopyranoside (IPTG) at a final concentration of 0.2 mM for 4 h at 30 °C. The cultured cells were harvested via centrifugation and sonicated in the 100 mM phosphate buffer (pH 7.8), containing 20 mM imidazole and 6 M guanidine hydrochloride. The clarified lysate was loaded on a column packed with Ni-NTA Sepharose (GE Healthcare). The recombinant protein was eluted with the buffer containing 0.5 M imidazole. The eluate was acidified with concentrated hydrochloric acid, and the fusion protein was cleaved by excess of CNBr over methionine at 25 °C for 18 h in the dark. The lyophilized products of the cleavage reaction were dissolved in water and loaded on a semi-preparative Reprosil-pur C_18_-AQ column (10 × 250 mm, 5-μm particle size, Dr. Maisch GmbH). Reversed-phase high-performance liquid chromatography (RP-HPLC) was performed with a linear gradient of acetonitrile in water containing 0.1% TFA. The peaks were monitored at 214 and 280 nm, collected, and analyzed by MALDI-TOF MS using Reflex III mass-spectrometer (Bruker Daltonics). The fractions with corresponding molecular masses (refer to supporting information, Table S1) were dried in vacuo and dissolved in water. The peptide concentrations were estimated using UV-absorbance and 280 nm (Implen NP80UV-vis Spectrophotometr, Germany). Recombinant buforin II was obtained as described previously [31]. Human cathelicidin LL-37 (>98% pure) was synthesized using a standard solid-phase method provided by Dr. Maxim N. Zhmak. Melittin (>98% pure) was synthesized using a standard solid-phase method, which was provided by Dr. Sergey V. Sychev.

### 3.3. Antimicrobial Assay

The antimicrobial activity of the peptides was determined via the method of two-fold serial dilutions in sterile 96-well flat-bottom polystyrene microplates (Eppendorf #0030730011) in Mueller–Hinton broth (MHB, Sigma, Kawasaki, Kanagawa, Japan) supplemented with 0.9% NaCl [32]. Bacterial cultures were grown in MHB at 37 °C until the culture reached an optical density OD_600_ of 1.0 and diluted with the broth so that to reach a final cell concentration of 10^6^ CFU/mL. Then 50 μL of the test culture were added to 50 μL of aqueous solutions of the peptides, previously diluted in 0.1% sterilized bovine serum albumin (BSA) to reduce non-specific binding of the peptides to the surface of the plates. Next, the plate was incubated for 24 h at 37 °C and 950 rpm on the plate thermoshaker (Biosan). The values of the minimum inhibitory concentrations (MIC) were determined as the minimum concentration of the peptide, at which there is no culture growth. The results were expressed as the median values of three independent experiments performed in triplicate.

### 3.4. Hemolytic Assay

The hemolytic activity of compounds was performed using human red blood cells (hRBS) of healthy male donor. Fresh blood was centrifuged to separate the erythrocyte fraction for 15 min at 500 *g* and washed three times with cold phosphate-buffered saline (PBS) (pH 7.4), after which an 8% (*v*/*v*) suspension of erythrocytes in PBS was prepared. Then, 50 µL of erythrocyte suspension was added to the test peptides, previously were dissolved in 50 µL of 0.1% BSA. The plate was incubated for 1.5 h at 37 °C and 950 rpm on the plate thermoshaker. After incubation, the plates were centrifuged for 15 min at 1000 *g* to settle intact erythrocytes. Next, aliquots of the supernatant were transferred to another plate to measure the amount of hemoglobin release by the absorbance of the solution at 405 nm. As “zero lysis” we used the supernatant obtained after centrifugation of erythrocytes incubated in a PBS solution without the addition of a peptide. As “100% lysis”, we used the supernatant obtained after centrifugation of erythrocytes incubated in a 0.1% Triton X-100, causing their complete lysis. The assays were conducted three times. The quantitative data were presented as an average means with standard deviations.

$$\mathrm{Hemolysis}\left(\%\right)=\frac{{\mathrm{OD}}_{405}\left(\mathrm{compound}\right)-{\mathrm{OD}}_{405}\left(\mathrm{zero}\mathrm{lysis}\right)}{{\mathrm{OD}}_{405}\left(100\%\mathrm{lysis}\right)-{\mathrm{OD}}_{405}\left(\mathrm{zero}\mathrm{lysis}\right)}\times 100\%$$

### 3.5. Cytotoxic Assay

The mammalian cell line was used in this study: transformed human embryonic kidney cells (HEK293T) which were obtained from a Russian bank of cell lines (Institute of Cytology RAS, Saint-Petersburg, Russia). The cytotoxic effect of AMP on mammalian cells was tested using the MTT assay. The technique is based on the ability of live cell dehydrogenases to reduce 3-(4,5-dimethylthiazol-2-yl)-2,5-diphenyl-2H-tetrazolium bromide (MTT reagent) to a water-insoluble violet crystalline formazan. Here, 10^4^ cells per well of transformed human embryonic kidney cells (HEK293T) in Dulbecco’s modified Eagle’s medium (DMEM/F12), supplemented with 10% fetal bovine serum (FBS), were placed into 96-well plates for 24 h in the CO_2_-incubator (5% CO2, 37 °C). Next, the culture liquid was replaced with fresh medium, in which the tested peptides were previously dissolved. After incubation for 16 h under these conditions, 20 µL of a solution of MTT in PBS (5 mg/mL) was added to each well, after which the incubation was continued for 4 h. The medium was carefully removed, 100 µL of a mixture of dimethyl sulfoxide (DMSO) and ethanol (1:1) was added to the wells to dissolve the formazan crystals, and the optical density of the solutions was measured at 570 nm using a microplate reader AF2200 (Eppendorf, Germany). Optical density in the wells containing cells cultured without the peptides was assumed to represent 100% cell viability. The experiments were carried out two times.

### 3.6. Assessment of Bacterial Membrane Permeabilization

The state of the cytoplasmic membrane of *E. coli* ML-35p was assessed by its permeability in the presence of AMP for n-nitrophenyl-β-D-galactopyranoside (ONPG), a substrate of β-galactosidase. As a result of the enzymatic cleavage of the substrate, the absorption of the solution in the well at a wavelength of 405 nm (ONPG hydrolysis product) increases. The bacterial test culture was grown in the trypticase soy broth (TSB) at 37 °C to stationary growth phase, washed three times with PBS (pH 7.4), and then dissolved in PBS (pH 7.4) to the final concentration of 2 × 10^7^ CFU/mL. The final concentration of OPNG was 2.5 mM and 100 mM NaCl was added. Assays were performed at 35 °C within 450 min under stirring at 300 rpm using microplate reader AF2200 (Eppendorf, Germany). Two independent experiments were performed, and the curve pattern was similar.

### 3.7. Resistance Induction Assay

The ability of peptides to induce bacterial resistance was assessed by the method of two-fold serial dilutions; this test was repeated every day. A culture of *E. coli* MDR Cl 1057 in the stationary phase was used as a starting inoculant and diluted with fresh MHB complemented with 0.9% NaCl to reach a final cell concentration of 10^6^ CFU/mL. Then, 50 μL of the obtained suspension was added to 50 μL of test drug solutions (initial MIC value was determined after incubation for 24 h at 37 °C). Next, on the second day, 2 μL of the bacterial suspension was taken from the well containing the sub-inhibitory concentration of the drug as close as possible to the MIC and diluted with 1 mL of the same media. Bacteria capable of growing at the maximum possible concentration of the peptides were passaged for three days on drug-free agar plates in the absence of an antibacterial agent, after which the final MIC values were determined.

### 3.8. Stability in Serum

The serum stability of peptides was determined in 25% human serum in PBS (pH 7.4) from healthy male donor as described previously [31]. Briefly, 20 mL of an aqueous peptide stock solution (3 mg/mL) was added to 480 µL of serum solution and incubated for: 2, 8 and 24 h at 37 °C. After incubation, serum proteins were selectively precipitated from the mixture by adding 10% TFA in the presence of 3 M urea. In the control samples, the precipitation was carried out immediately after the addition of serum. Next, the samples were stored at 0 °C for 30 min and centrifuged at 14,000× *g* for 20 min. The obtained supernatants were analyzed by analytical RP-HPLC (Symmetry 300 C18 column) and MALDI-TOF MS. The amount of the intact peptide was estimated from the height of the corresponding peak on the RP-HPLC chromatogram. Two independent experiments in duplicate were performed for each peptide.

### 3.9. Measurement of Anti-Biofilm Activity

The ability of peptides to influence the formation of biofilms was evaluated by the method of two-fold serial dilutions in a liquid nutrient medium [26]. Bacterial culture was grown for 24 h and diluted with the LB medium to final concentration of 10^6^ CFU/mL. The tested peptides were prepared in sterile water in a volume of 50 μL. Then, 50 μL of bacterial suspension was added to the aqueous solution of peptides. After 24 h of incubation at 32 °C and stirred at 120 rpm, the MIC values were determined as the minimum concentrations of compounds at which planktonic bacterial growth were inhibited by more than 90% spectrophotometrically at 570 nm. Next, the medium was removed, wells were rinsed with sterile water three times, and 0.1% aqueous solution of crystal violet (CV, Sigma, Kawasaki, Kanagawa, Japan) was added for 40 min at 25 °C. Then, the liquid was removed, and wells were washed with sterile water to clean up the excess dye and treated 30% acetic acid was added to each well and incubated at 25 °C for 15 min for the extraction of CV from biofilms. Further, the extracts were transferred to a new plate and absorption was measured at 570 nm. This experiment was carried out in triplicate.

### 3.10. The Effect of Peptides on Established Biofilms

The effect of peptides on pre-formed biofilms was evaluated as follows. Biofilms were grown on a 96-well plate at 32 °C for 24 h and stirred at 120 rpm to ensure biofilm formation primarily on the wall of the wells. The ability of peptides to eradicate mature biofilm after 2-h exposure was monitored using the dying method by CV solution (0.1%), as described above. Bacterial survival in the biofilms after 2-h exposure was evaluated via a MTT assay after incubation with a tested peptide. Then, wells were rinsed with sterile water, treated with an MTT solution (Sigma, Kawasaki, Kanagawa, Japan) in PBS, and incubated for 4 h at 37 °C. Next, the medium was removed. The resulting formazan crystals were dissolved in a mixture of EtOH and DMSO (1:1) for 10 min. The optical density of the solution was measured at 570 nm. Bacterial survival was calculated as percent relative to samples without antimicrobial agent. All data were obtained from at least three independent experiments in 3 repetitions.

### 3.11. Animal Studies

For animal experiments, peptides Ap9, Ap11, UuBRI-21, and AA139 were produced in endotoxin-free ClearColi^®^ BL21(DE3) expression system according to the protocol presented in the Section 3.2.

Experiments were performed with female 8–10-week-old BALB/c mice (22–24 g, “Andreevka” laboratory animal nursery FMBA, Russia). Compared to males, female mice are not prone to aggressive behavior and any social contacts with each other while in the same cage. Therefore, the use of females minimizes the risk of secondary infection of animals during the experiment and increases the reliability of the initial data on therapeutic efficacy. All animal experiments were performed at the State Research Center for Applied Microbiology and Biotechnology (SRCAMB) in Obolensk, Russia. The animals were housed in groups of 5 in polycarbonate cages. Food and water were provided ad libitum.

The peritonitis in vivo model was performed using an *E. coli* 3421/E19 strain in BALB/c mice that were rendered neutropenic by injecting 0.5 mL cyclophosphamide (Sigma, Kawasaki, Kanagawa, Japan) solution intraperitoneally (i.p.) for 4 days (200 mg/kg) and 1 day (100 mg/kg) prior to inoculation of bacteria. *E. coli* 3421/E19 (strain ID B-8871 in the State Collection of Pathogenic Microorganisms “Obolensk”) is a virulent tetracycline-resistant strain having a hemolytic activity. At 1 h, mice were inoculated i.p. with 0.5 mL of *E. coli* 3421/E19 suspension (1 × 10^6^ CFU per animal). Mice were then treated intravenously (i.v.) with a single 10 mg/kg dose of a peptide (Ap9, Ap11, UuBRI-21, or AA139), polymyxin B (Applichem, 5 mg/kg) or control (saline) at 1 h (*n* = 5 mice per group). CFU counts were determined from blood and peritoneal fluid (PF) 5 h after treatment. The mice were euthanized by CO_2_ asphyxiation and blood was collected by axillary cut down. A total of 2 mL sterile saline was injected i.p. and the abdomen gently massaged before it was opened, fluid sampled with a pipette, serially diluted and placed on Endo agar supplemented with 20 µg/mL of tetracycline for CFU identification.

*In vivo* efficacy of peptides was tested in the mice septicemia model induced by *E. coli* ATCC 25922. Mice were infected with 0.5 mL of bacterial suspension (1 × 10^6^ CFU per animal) in PBS (pH 7.2) with 2.5% mucin (*w*/*v*) via i.p. injection. A total of 4 groups with 8 animals were used. The first group received ciprofloxacin (Sigma, Kawasaki, Kanagawa, Japan) administered i.p. once (1 h post-infection) at a dose of 10 mg/kg. The second group received saline (as a vehicle control) administered i.p. once (1 h post-infection). Two other groups received abarenicin analog Ap9 or AA139 each administered i.p. two times (1 and 4 h post-infection) at a dose of 10 mg/kg. Survival was monitored for 7 days. After that survived animals were euthanized by CO_2_ asphyxiation. The spleen was aseptically removed, homogenized, serially diluted, and placed on Endo agar for CFU identification.

All experiments were approved by the Institutional Bioethic Committee of the SRCAMB and performed according to the Russian Federal rules and Directive 2010/63/EU of the European Parliament and of the Council.

### 3.12. Statistical Analysis

Statistical analysis was performed using Graphpad prism 6.0 software (GraphPad Software Inc.), and a value of *p* < 0.05 was considered statistically significant. All comparisons were based on mean ± standard deviation of the mean (SD) of three independent experiment.

## 4. Conclusions

In this paper, two novel BRICHOS-related β-hairpin antimicrobial peptides, abarenicin, and UuBRI-21 from the marine polychaeta *Abarenicola pacifica* and *Urechis unicinctus*, respectively, were obtained and investigated. Considering the stronger antibacterial potential and a low ability of abarenicin to be bound by components of serum, this peptide was selected for further modification. Rational design based on individual hydrophobic amino acid substitutions in the structure of abarenicin-1 led us to Ap9 (Y8A, M9R) and Ap11 (Y8A, M9R, V13A) analogs with an enhanced bacterial cell selectivity. Surprisingly, the variant Ap11 was found to be ineffective in the treatment of peritonitis in mice model, although having similar changes in the β-turn region as compared with AA139 which is known to be highly effective in vivo. However, Ap9 demonstrated a promising efficacy in *E. coli* infection in vivo models. In addition, abarenicin analogs did not significantly induce bacterial resistance and exhibited antibiofilm activity against both emerging and mature biofilms. Considering a high antibacterial and antibiofilm potency in vitro towards different strains of *P. aeruginosa*, we assessed an efficacy of Ap9 in animal models of this bacterial infection. Despite the fact that the analog Ap9 has a comparable in vitro and in vivo biological activity profile with AA139, additional animal studies examining pharmacokinetics and toxicity are required to enter the preclinical stage. In summary, the abarenicin analog Ap9 is a new attractive drug candidate treatment of diseases caused by Gram-negative bacterial pathogens.

## Figures and Tables

**Table 1:** Influence of FBS in the medium on antibacterial activity of the novel peptides against *E. coli* ML-35p.

Peptide	Minimum Inhibitory Concentration, µM	
	MHB + 0.9% NaCl	MHB + 0.9% NaCl + 25% FBS
Abarenicin	0.25	1
AA139	0.25	1
UuBRI-21	0.25	4

## Data Availability

Not applicable.

## Supplementary Materials

The following supporting information can be downloaded at: https://www.mdpi.com/article/10.3390/md20080517/s1. Figure S1: Amino acid sequence alignment of the precursors of arenicin-like BRICHOS-related AMPs; Table S1: Characteristics of the recombinant AMPs.
