# Supplementary material for: Novel β-Hairpin Peptide from Marine Polychaeta with a High Efficacy against Gram-Negative Pathogens
Source: Mar Drugs. 2022 Aug 13;20(8):517. doi: 10.3390/md20080517 (PMC9410094; doi:10.3390/md20080517)
Supplement: Supplementary file 1 [file marinedrugs-20-00517-s001.zip › marinedrugs-1869413-supplementary.pdf]

Supplementary Material

Novel β-Hairpin Peptide from Marine Polychaeta with a High Efficacy against Gram-Negative Pathogens

Victoria N. Safronova [1], Ilia A. Bolosov [1], Roman N. Kruglikov [1], Olga V. Korobova [2], Eugenia S. Pereskokova [2],
Alexander I. Borzilov [2], Pavel V. Panteleev [1,*] and Tatiana V. Ovchinnikova [1,3,*]

[1] M. M. Shemyakin & Yu. A. Ovchinnikov Institute of Bioorganic Chemistry, The Russian Academy of Sciences, Miklukho-Maklaya str., 16/10, 117997 Moscow, Russia;

[2] State Research Center for Applied Microbiology & Biotechnology (SRCAMB), 142279 Obolensk, Russia

[3] Department of Bioorganic Chemistry, Faculty of Biology, Lomonosov Moscow State University, 119234 Moscow, Russia

* Correspondence: ibch@inbox.ru (P.V.P.); ovch@ibch.ru (T.V.O.)

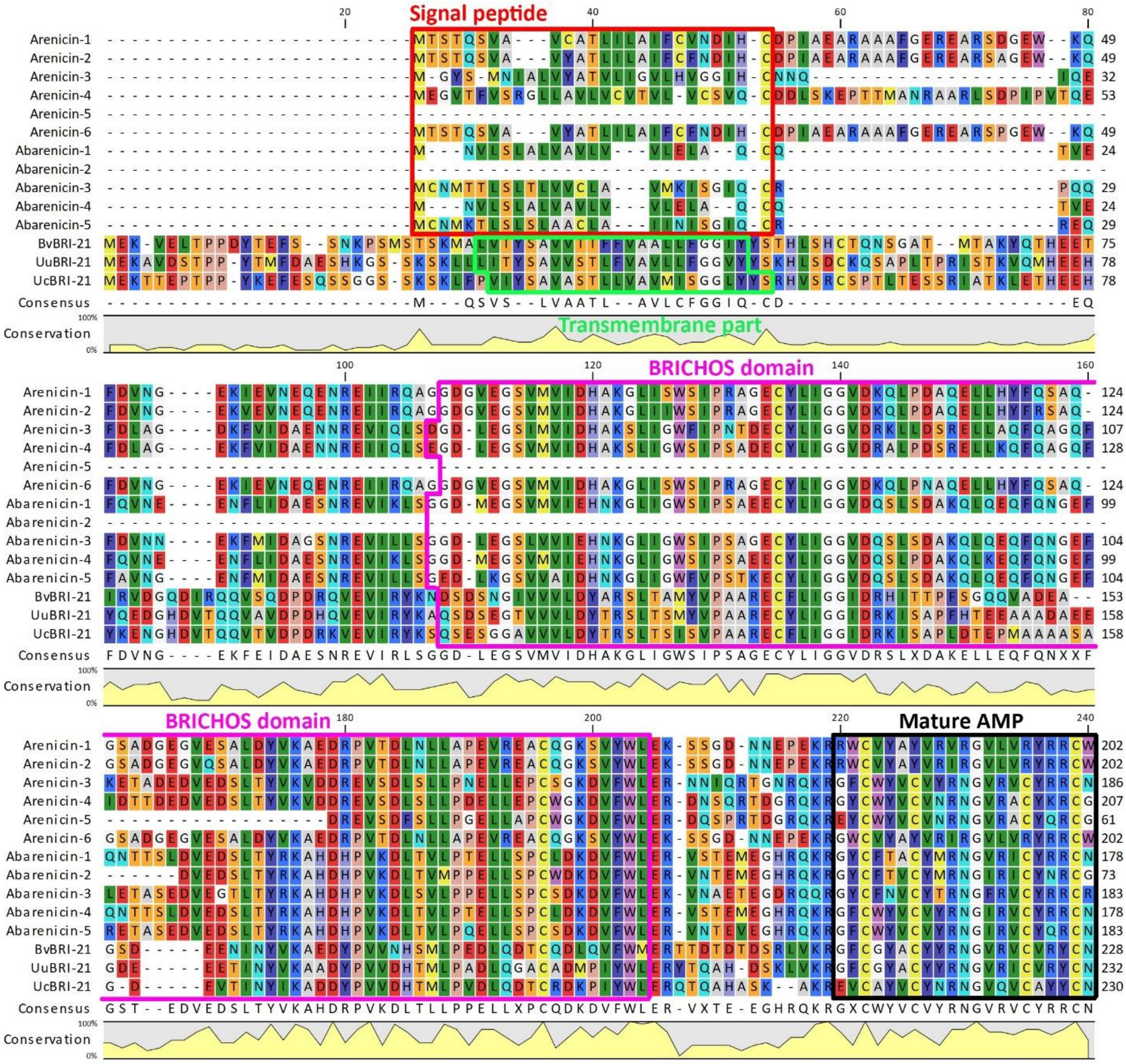

Figure S1. Amino acid sequence alignment of the precursors of arenicin-like BRICHOS-related AMPs. The alignment was constructed using CLC Sequence Viewer software (version 8.0). Signal peptide sequence identified with SignalP 5.0 (https://services.healthtech.dtu.dk/service.php?SignalP-5.0), transmembrane parts identified with TMHMM - 2.0 (https://services.healthtech.dtu.dk/service.php?TMHMM-2.0), and BRICHOS domain sequence identified with MyHits Motif Scan (https://myhits.isb-sib.ch/cgi-bin/motif_scan) are highlighted with red, green, and purple boxes, respectively. Mature peptide sequences are highlighted with black box.

Table S1. Characteristics of the recombinant AMPs.

Peptide	Hydrophobicity Index [1]	RP-HPLC Retention Time, min [2]	Recombinant Peptide Final Yield, mg/L	Calculated [M+H]+ Monoisotopic Mass, Da [3]	Measured Monoisotopic m/z Value [4]
Abarenicin*	-0.157	25.77	5.1	2527.16	2527.04
Ap1	-0.010	26.05	5.9	2435.13	2434.92
Ap2	-0.310	24.29	6.2	2520.20	2520.20
Ap3	-0.252	25.47	5.1	2485.11	2484.82
Ap4	-0.552	24.79	8.4	2570.18	2570.26
Ap5	-0.271	25.53	4.4	2499.13	2498.85
Ap6	-0.571	24.69	7.2	2584.19	2584.11
Ap7	-0.381	25.61	8.2	2485.11	2485.10
Ap8	-0.681	24.34	11.2	2570.18	2570.07
Ap9	-0.405	25.37	5.8	2478.15	2478.11
Ap10	-0.667	24.41	8.2	2542.15	2542.00
Ap11	-0.519	25.17	4.9	2450.12	2450.03
UuBRI-21	0.029	28.48	7.1	2476.08	2476.26
AA139	0.429	25.67	7.2	2549.17	2549.95

[1] Mean Kyte–Doolittle hydrophobicity index (GRAVY) was calculated using the Expasy ProtParam tool. The maximum and minimum values of this index are +4.5 and -4.5 for poly-Ile and poly-Arg sequences, respectively. [2] Retention times were measured using semipreparative reversed-phase high-performance liquid chromatography (RP-HPLC) on a C18 column with a linear gradient from 5 to 80% (*v/v*) acetonitrile in water containing 0.1% trifluoroacetic acid (TFA) within 50 minutes. [3] Molecular masses were calculated by considering the presence of four Cys residues forming two disulfide bonds. [4] Molecular masses were determined experimentally using MALDI-TOF mass spectrometry. * - abarenicin-1[M9L]

Table S2. The data on analyzed transcriptomes from polychaeta

Animal	Accession number	# of reads	Sequencing platform	Sample information
Abarenicola pacifica	SRX7658526	35,669,605	Illumina HiSeq 2000	Whole animal, mature
Arenicola marina	SRX1015734	68,660,484	Illumina HiSeq 1000	Whole animal, mature
Bonellia viridis	SRX1024222	45,343,168	Illumina HiSeq 2000	Whole animal, mature
Urechis unicinctus	SRX4526081	27,595,395	Illumina HiSeq X Ten	Whole animal, larvae
